# Delta De Ritis Ratio Is Associated with Worse Mortality Outcomes in Adult Trauma Patients with Moderate-to-Severe Traumatic Brain Injuries

**DOI:** 10.3390/diagnostics12123004

**Published:** 2022-12-01

**Authors:** Ching-Hua Tsai, Cheng-Shyuan Rau, Sheng-En Chou, Wei-Ti Su, Shiun-Yuan Hsu, Ching-Hua Hsieh

**Affiliations:** 1 Department of Trauma Surgery, Kaohsiung Chang Gung Memorial Hospital, Chang Gung University College of Medicine, Kaohsiung 83301, Taiwan; 2 Department of Neurosurgery, Kaohsiung Chang Gung Memorial Hospital, Chang Gung University College of Medicine, Kaohsiung 83301, Taiwan

**Keywords:** aspartate aminotransferase, alanine aminotransferase, De Ritis ratio, delta De Ritis ratio, mortality, traumatic brain injury

## Abstract

This study aimed to investigate whether changes in the De Ritis ratio (DRR) can be used to stratify the mortality risk of patients with moderate-to-severe traumatic brain injury (TBI). This retrospective study reviewed data for 1347 adult trauma patients (134 deaths and 1213 survival) with moderate-to-severe TBI between 1 January 2009, and 31 December 2020, from the registered trauma database. The outcomes of the patients allocated into the two study groups were compared based on the best Delta DRR (ΔDRR) cutoff point. The first and second DRR of patients who died were significantly higher than those of patients who survived. Elevation of DRR 72–96 h later was found for patients who died, but not for those who survived; the ΔDRR of the patients who died was significantly higher than that of those who survived (1.4 ± 5.8 vs. −0.1 ± 3.3, *p* = 0.004). Multivariate logistic regression analysis revealed that ΔDRR was a significant independent risk factor for mortality in these patients. Additionally, a ΔDRR of 0.7 was identified as the cutoff value for mortality stratification of adult trauma patients at high risk of mortality with moderate-to-severe TBI.

## 1. Introduction

Traumatic brain injury (TBI) is a common critical illness observed by trauma surgeons. There are 1.7 million estimated people in the United States who sustain TBIs each year, accounting for 4.8% of all injuries observed during visits to emergency departments [1]. Of these, 275,000 patients are hospitalized, and 52,000 have died, comprising approximately 30% of all injury deaths in the United States [1].

Potential interactions between brain injury and body organs have been reported [2]. In animal models, brain injury produces an inflammatory response in the circulatory and peripheral organs, especially in the liver [3,4,5]. An increase in the expression of acute-phase response proteins, chemokines, and several inflammatory mediators, as well as the accumulation of macrophages and dying cells in the liver, demonstrate hepatic inflammation after TBI [4]. One study reported that 6 h after the onset of TBI, serum levels of liver enzymes alanine aminotransferase (ALT) and aspartate aminotransferase (AST) increased in patients [6]. Another study reported that 12 and 24 h after the onset of TBI, the activity of serum AST and ALT increased with associated liver dysfunction [7]. Following TBI, ALT levels worsened and gradually resolved in patients [8].

Because ALT is predominantly found in the cytosol of hepatocytes, an increase in the level of serum ALT indicates parenchymal liver disease with liver-specific dysfunction. In contrast, an increase in the serum AST level is involved in a more systemic phenomenon other than parenchymal liver disease because AST is present in many organs other than the liver, including the brain, heart, kidneys, and skeletal muscle [9]. Metabolic disorders, ischemia-reperfusion injury, and oxidative stress may increase serum AST expression [10,11,12]. Because the AST level is associated with other organs affected systemically and ALT level specifically indicates parenchymal liver disease, changes in these two enzymes may be useful for the diagnosis or risk stratification of various illnesses [9]. Serum ratio of AST/ALT, the so-called “De Ritis ratio (DRR)”, has been reported to be a valuable marker in differentiating varying causes of liver disease [9,13,14], surrogating different kinds of malignancies [13,15,16,17,18,19,20,21], and provides valuable information on the risk assessment of patients with heart diseases [22,23,24], acute kidney injury [25,26,27], sepsis [28], and even patients with COVID-19 [29,30,31,32].

To investigate the relationship between DRR changes in patients with TBI and the mortality risk of patients with TBI, we proposed the delta DRR (ΔDRR), which is the change in DRR at the time of arrival to the emergency room and 72–96 h later. This study aimed to investigate whether ΔDRR is useful for stratifying the mortality risk of patients with TBI. Since mortality in patients with mild TBI is rare, this study focused on patients with moderate-to-severe TBI, and the primary outcome of this study was in-hospital mortality.

## 2. Materials and Methods

### 2.1. Study Population and Data Collection

**Figure 1:**
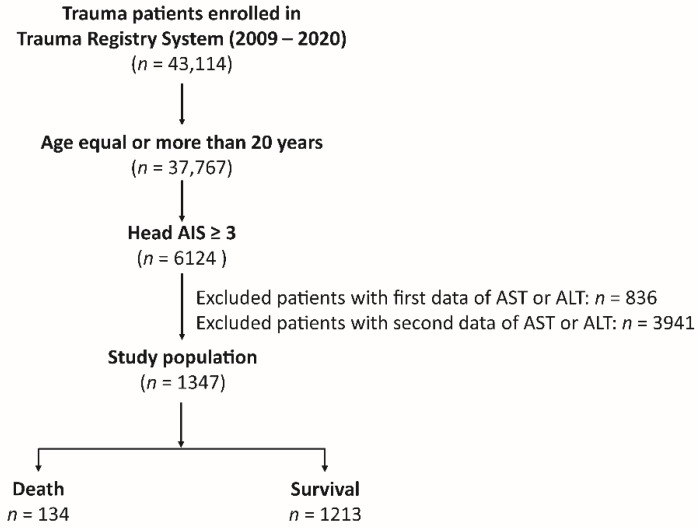
Flowchart illustrating the inclusion of hospitalized adult trauma patients with moderate-to-severe traumatic brain injuries from the registered trauma database. Those patients who lacked the first AST or ALT data upon arrival at the emergency room and at 72–96 h later, were excluded from the study population.

As shown in Figure 1, of the 43,114 hospitalized trauma patients with all trauma causes enrolled in the Trauma Registry System of the Chang Gung Memorial Hospital [33,34,35] between 1 January 2009 and 31 December 2020, 6124 adult patients aged ≥20 years with moderate-to-severe TBI, defined as a head abbreviated injury scale (AIS) ≥3, were included in our study. After excluding patients who lacked the first AST or ALT data upon arrival at the emergency room (*n* = 836) and those who lacked the second AST or ALT data 72–96 h later (*n* = 3941), 1347 adult trauma patients with moderate-to-severe TBI were included in the study population. The patients’ medical information was retrieved from the registered trauma database, including sex, age, serum AST and ALT levels (U/L) upon arrival at the emergency room. Within 72–96 h of arrival, diagnosis of traumatic brain injuries was conducted (subarachnoid hemorrhage (SAH), subdural hemorrhage (SDH), epidural hemorrhage (EDH), and intracerebral hemorrhage (ICH)) with an abbreviated injury scale ≥3, indicating a moderate-to-severe injury, pre-existing comorbidities, Glasgow Coma Scale (GCS) score, Injury Severity Score (ISS), and in-hospital mortality. The first and second DRR were calculated from the ratio of serum AST and ALT levels upon arrival at the emergency room and 72–96 h later, respectively. The delta DRR was calculated as the second DRR minus the first DRR.

### 2.2. Statistical Analyses

Statistical analyses were performed using SPSS (version 23.0; IBM Inc., Chicago, IL, USA). Normally distributed continuous data were analyzed using the Kolmogorov–Smirnov test. Continuous data with normal distribution were analyzed using analysis of variance with Bonferroni post-hoc correction, whereas non-normally distributed continuous data were compared using the Mann–Whitney U test. Continuous and non-continuous data were expressed as mean ± standard deviation or median with interquartile range (IQR) between Q1 and Q3, respectively. Categorical data were compared using two-sided Fisher’s exact test or Pearson’s χ^2^ test. Univariate predictive variables resulting in patient mortality were analyzed using multivariate logistic regression analysis to identify the independent risk factors for mortality, presenting odds of risk as odds ratios (ORs) and 95% confidence intervals (CIs). The predictive performance of ΔDRR for patient mortality was determined based on the area under the receiver operating characteristic curve (AUC). The best cut-off point was derived from receiver operating characteristic curves based on the maximal Youden index; a value determined using sensitivity + specificity − 1. The best cutoff point reflected the maximal correct classification accuracy when plotting receiver operating characteristic (ROC) curves. Comparison of the outcomes of the patients allocated into two groups of the study population based on the best cutoff point of a ΔDRR value was performed by presenting an adjusted odds ratio (AOR) of mortality, which was calculated with logistic regression under the control of variables with significant differences in patients’ injury characteristics. A two-tailed *p* value of <0.05 was considered significant for all analyses.

## 3. Results

### 3.1. Injury and Patient Characteristics

There were 134 dead and 1213 surviving patients in this study. As shown in Table 1, there were no significant differences in sex between the dead and surviving patient groups. Patients who died were significantly older than those who survived (*p* < 0.001). There was a significantly higher rate of sustaining SDH in the dead than in surviving patients (69.4% vs. 60.1%, *p* = 0.036). However, there was no significant difference in the incidence of SAH, EDH, or ICH between patients who died and those who survived. Regarding liver enzymes, there were no significant differences in the first AST level between the dead and surviving patients (81.1 Â ± 84.1 Â U/L vs. 97.2 ± 217.0 U/L, *p* = 0.395), but there was a significantly lower level of the first ALT in the dead patients than that in the surviving patients (46.6 ± 53.2 vs. 62.0 ± 98.7 U/L, *p* = 0.005); however, there was a significantly higher level of the second AST in the dead than in the surviving patients (167.0 ± 411.5 vs. 55.3 ± 98.3 U/L, *p* = 0.002), but no significant difference in the second ALT level between the two groups of patients (173.6 ± 720.7 vs. 51.4 ± 175.3 U/L, *p* = 0.053). The first and second DRR of the patients who died were significantly higher than those of the surviving patients (first measurement: 1.9 ± 0.8 vs. 1.6 ± 0.7, *p* < 0.001; second measurement: 3.3 ± 5.9 vs. 1.6 ± 3.3, *p* = 0.001). These results imply that an elevated DRR in the second measure of those death patients was mostly attributed to a higher AST level. Furthermore, in comparison with the DRR upon arrival at the emergency room, elevation of DRR 72–96 h later was found for those patients who died, while the values remained at similar levels for the surviving patients; therefore, the ΔDRR of the patients who died was significantly higher than that of the patients who survived (1.4 ± 5.8 vs. −0.1 ± 3.3, *p* = 0.004). Significantly higher rates of pre-existing comorbidities such as cerebrovascular accidents (CVA), hypertension (HTN), coronary artery disease (CAD), and end-stage renal disease (ESRD) were found in patients who died than in those who survived. The deceased patients presented with a significantly lower GCS but higher ISS than the surviving patients (median [IQR, Q1–3], GCS:7 [3–15] vs. 13 [7–15], *p* < 0.001; ISS:25 [20–31] vs. 20 [16–25], *p* < 0.001). 

The comparison between 437 female and 910 male patients in the study population (Table 2) revealed that there was no significant difference in the first and second DRR, ΔDRR, or mortality between female and male patients. However, female patients were significantly older, had higher GCS scores, and had sustained less severe injuries than male patients.

### 3.2. Analysis of the Risk Factors for Mortality

Univariate analysis revealed that age, presence of CVA, HTN, CAD, ESRD, GCS score, ISS, and ΔDRR were significant risk factors for mortality in adult trauma patients with moderate-to-severe TBI (Table 3). Subsequent multivariate logistic regression analysis revealed that age (OR, 1.03; 95% CI, 1.02–1.04; *p* < 0.001), existence of CVA (OR, 2.20; 95% CI, 1.05–4.62; *p* = 0.037), ESRD (OR, 5.51; 95% CI, 2.48–12.25; *p* < 0.001), GCS (OR, 0.87; 95% CI, 0.83–0.91; *p* < 0.001), ISS (OR, 1.07; 95% CI, 1.04–1.09; *p* < 0.001), and ΔDRR (OR, 1.04; 95% CI, 1.01–1.08; *p* = 0.021) were significant independent risk factors for mortality in these patients.

### 3.3. Analysis of the Plotted ROC Curve

**Figure 2:**
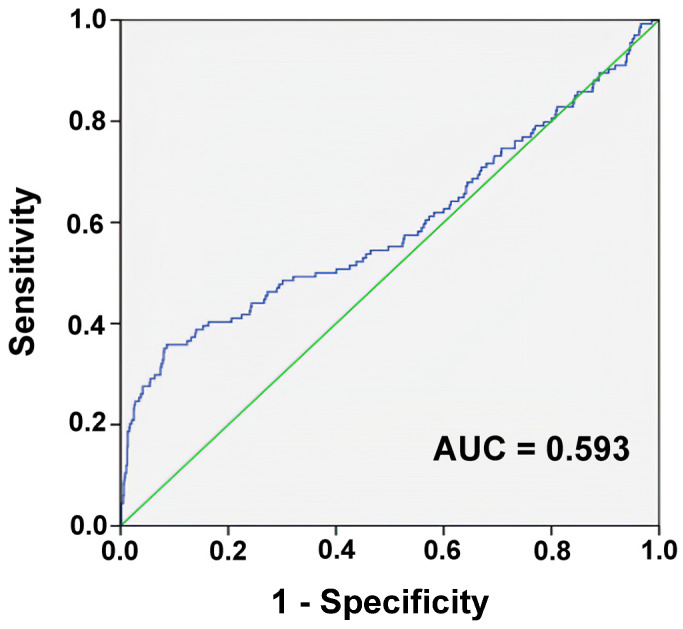
Receiver operating characteristic curves and area under the curve (AUC) of the delta De Ritis ratio for predicting the mortality of the adult trauma patients with moderate-to-severe traumatic brain injuries.

According to the ROC curve analysis, a ΔDRR of 0.7 as the cutoff point had the highest AUC of 0.593, with a sensitivity of 0.358 and specificity of 0.913 (Figure 2). The accuracy of the discriminating power of ΔDRR alone in predicting patient mortality was low.

### 3.4. Comparison of the Outcomes of Patients with ΔDRR ≥ 0.7 vs. Those with ΔDRR < 0.7

As shown in Table 4, there was no significant difference in sex between patients with ΔDRR ≥ 0.7 vs. those ΔDRR < 0.7. The patients with a ΔDRR ≥ 0.7 were significantly older than those with a ΔDRR of <0.7 (*p* < 0.001). There was a significantly higher rate of sustaining SDH in patients with a ΔDRR ≥ 0.7 than those <0.7 (70.6% vs. 59.8%, *p* = 0.010). However, there was no significant difference in the incidence of SAH, EDH, or ICH between patients with a ΔDRR ≥ 0.7 and <0.7. The first DRR of these two groups of patients were not significantly different (1.7 ± 0.6 vs. 1.7 ± 0.7, *p* = 0.961); however, the second DRR of the patients with a ΔDRR of ≥0.7 was significantly higher than those with a ΔDRR of <0.7 (5.2 ± 10.2 vs. 1.3 ± 0.6, *p* < 0.001). The elevation of ΔDRR was mostly attributed to an elevated AST level in the second measurement, as the ALT level in the second measurement was not significantly different between these two groups of patients, and the first DRR of these two groups of patients was not significantly different. Significantly higher rates of pre-existing comorbidities of HTN, congestive heart failure, and ESRD were found in patients with a ΔDDR ≥0.7 vs. those with a ΔDRR < 0.7. Patients with a ΔDRR of ≥0.7 presented with a significantly lower GCS, but higher ISS than those with a ΔDRR of <0.7 (median [IQR, Q1–3], GCS:11 [6–15] vs. 13 [7–15], *p* = 0.031; ISS: 25 [16–29] vs. 20 [16–25], *p* = 0.001). Patients with a ΔDRR of ≥0.7 presented with a significantly higher mortality rate than patients with a ΔDRR of <0.7 (31.4% vs. 7.2%, OR 95% CI: 5.89 (3.93–8.84), *p* < 0.001). Under the control by sex, age, CVA, ESRD, GCS, and ISS, the patients with a ΔDRR of ≥0.7 still presented with a significantly higher adjusted mortality rate than the patients with a ΔDRR of <0.7 (AOR, 95% CI: 4.21 (2.68–6.63), *p* < 0.001).

## 4. Discussion

Aspartate aminotransferase and ALT catalyze nucleotide and nonessential amino acids involved in aerobic glycolysis, and function as important links between protein and carbohydrate metabolism [36,37,38]. In contrast to the role of ALT in the glucose-alanine cycle to produce glucose to cope with sugar consumption [9], AST plays a vital role in the malate–aspartate shuttle pathway involved in aerobic glycolysis, which allows conversion between NADH and NAD+ [39]. Alanine aminotransferase is mainly present in the cytoplasm, whereas AST is found not only in the cytoplasm but also in the mitochondria [9,40,41]. An isolated elevation in AST levels suggests a non-hepatic source of AST, which is usually due to injury to non-liver cells, particularly cells that contain mitochondria [9]. Elevated DRR levels may indicate dysfunction at the mitochondrial level, which can lead to increased oxidative stress [9,42,43]. It has been reported that DRR is related to tumor metabolism in many malignancies utilizing glucose [44]. This study revealed that ΔDRR was a significant independent risk factor for mortality in patients with moderate-to-severe TBI, and a ΔDRR ≥ 0.7 was associated with a higher risk of mortality in the patients. Moreover, the elevation in the ΔDRR value was mostly attributed to an elevated AST level in the second measurement. Therefore, an elevated DRR may reflect adapted conditions of trauma patients under stress to cope with glucose consumption. However, further investigation is required to verify this hypothesis.

A single measurement of DRR as a predictive biomarker may vary greatly depending on the disease studied. For example, a DRR of ≥1.19 in patients who underwent heart surgery for valve replacement and warfarin treatment was significantly associated with bleeding tendency [45]. A DRR of ≥1.2 specifies a higher mortality risk for patients with acute myocardial infarction [22]. A DRR of ≥1.5 and 1.6 is a significant prognostic factor for patients with renal cell carcinoma and upper urinary tract urothelial carcinoma, respectively, following surgical treatment [46]. A DRR of >1.67 is associated with a two-fold OR for critical limb ischemia in patients with peripheral arterial occlusive disease [47]. A DRR > 2.0 is used as a prognostic indicator for patients with distal cholangiocarcinoma [48]. In this study, the first measurement of the DRR of patients who died upon arrival at the emergency room was significantly higher than that of surviving patients (1.9 ± 0.8 vs. 1.6 ± 0.7, *p* < 0.001), which may be related to a pathophysiological response or a baseline difference in liver function between patients who died and survived. However, differentiating between these two mechanisms is difficult and it is impossible to design a retrospective study. Liver function may be associated with the outcome of patients with TBI; for example, liver cirrhosis is associated with coagulopathy and presents as a poor comorbidity factor in patients with TBI [49]. Considering that the baseline liver function may be different and elevation of DRR at 72–96 h was later found in patients who died, rather than in those who survived, the measurement of ΔDRR may provide more valuable information for risk stratification. In this study, a ΔDRR of 0.7 was identified as the cutoff value for mortality stratification. 

This study has some limitations: First, selection bias may have occurred because of the retrospective design of this study, thus precluding further investigation of the circulating biomarkers and findings in neuroimaging of traumatic brain injuries with the expression of as well as the change in DRR. Second, the assessment of in-hospital mortality, but not the death declared upon arrival at the emergency room, and long-term mortality, which is lacking in the registered trauma database, may lead to a selection bias in the assessment of the outcome. Furthermore, the exclusion of patients without AST and ALT data may have resulted in selection bias. Third, interventions such as resuscitation, blood transfusion, and surgery could have led to different outcomes in the studied patients; however, we can only assume that the outcomes of these interventions were uniform across the studied population. Fourth, DRR may differ in the presence of undetected liver disease, use of drugs that can disturb the levels of AST or ALT in circulation, and interventions such as prehospital resuscitation. Moreover, both serum AST and ALT increase with body weight, but such increase is more prominent for ALT than AST [50]; therefore, the difference in body weight may result in bias in the presentation of the DRR of the patients. Finally, the study population was limited to a single urban trauma center, which limits the generalizability of the results to other regions. 

## 5. Conclusions

This study revealed that a significant elevation of DRR at 72–96 h after arrival at the emergency room was found in patients who died, but not in surviving patients. A ΔDRR of 0.7 may provide a cutoff value for stratification of adult trauma patients at high risk of mortality with moderate-to-severe TBI.

## Figures and Tables

**Table 1:** Comparison of the injury and patient characteristics of death and survival patients in the study population.

Variables	Death *n* = 134	Survival *n* = 1213	OR (95% CI)	*p*
Female, n (%)	46 (34.3)	391 (32.2)	1.10 (0.75–1.60)	0.623
Age, years	62.4 ± 18.7	56.0 ± 19.3	—	<0.001
Diagnosis				
SAH, n (%)	52 (38.8)	546 (45.0)	0.78 (0.54–1.12)	0.170
SDH, n (%)	93 (69.4)	729 (60.1)	1.51 (1.03–2.21)	0.036
EDH, n (%)	16 (11.9)	193 (15.9)	0.72 (0.42–1.24)	0.228
ICH, n (%)	44 (32.8)	353 (29.1)	1.19 (0.81–1.74)	0.368
De Ritis ratio (1st)	1.9 ± 0.8	1.6 ± 0.7	—	<0.001
AST, (U/L)	81.1 ± 84.1	97.2 ± 217.0	—	0.395
ALT, (U/L)	46.6 ± 53.2	62.0 ± 98.7	—	0.005
De Ritis ratio (2nd)	3.3 ± 5.9	1.6 ± 3.3	—	0.001
AST, (U/L)	167.0 ± 411.5	55.3 ± 98.3	—	0.002
ALT, (U/L)	173.6 ± 720.7	51.4 ± 175.3	—	0.053
ΔDDR	1.4 ± 5.8	–0.1 ± 3.3	—	0.004
Comorbidities				
CVA, n (%)	12 (9.0)	55 (4.5)	2.07 (1.08–3.97)	0.025
HTN, n (%)	58 (43.3)	410 (33.8)	1.50 (1.04–2.15)	0.029
CAD, n (%)	17 (2.7)	76 (6.3)	2.17 (1.24–3.80)	0.005
CHF, n (%)	2 (1.5)	7 (0.6)	2.61 (0.54–12.70)	0.217
DM, n (%)	29 (21.6)	219 (18.1)	1.25 (0.81–1.94)	0.309
ESRD, n (%)	14 (10.4)	23 (1.9)	6.04 (3.03–12.04)	<0.001
GCS, median (IQR)	7 (3–15)	13 (7–15)	—	<0.001
3–8	81 (60.4)	371 (30.6)	3.47 (2.40–5.01)	<0.001
9–12	12 (9.0)	210 (17.3)	0.47 (0.26–0.87)	0.013
13–15	41 (30.6)	632 (52.1)	0.41 (0.28–0.60)	<0.001
ISS, median (IQR)	25 (20–31)	20 (16–25)	—	<0.001
1–15	4 (3.0)	112 (9.2)	0.30 (0.11–0.83)	0.014
16–24	33 (24.6)	633 (52.2)	0.30 (0.20–0.45)	<0.001
≥25	97 (72.4)	468 (38.6)	4.17 (2.81–6.20)	<0.001

ALT, alanine aminotransferase; AST, aspartate aminotransferase; CAD, coronary artery disease; CHF, congestive heart failure; CI, confidence interval; CVA, cerebral vascular accident; ΔDDR, delta De Ritis ratio (i.e., 2nd De Ritis ratio minus 1st De Ritis ratio); DM, diabetes mellitus; EDH, epidural hemorrhage; ESRD, end-stage renal disease; GCS, Glasgow Coma Scale; HTN, hypertension; ICH, intracerebral hemorrhage; IQR, interquartile range; ISS, injury severity score; OR, odds ratio; SAH, subarachnoid hemorrhage; SDH, subdural hemorrhage.

**Table 2:** Comparison of the injury characteristics in male and female patients.

Variables	Female *n* = 437	Male *n* = 910	OR (95% CI)	*p*
Age, years	61.9 ± 18.1	54.1 ± 19.4	—	<0.001
De Ritis ratio (1st)	1.7 ± 0.7	1.7 ± 0.8	—	0.385
AST, (U/L)	94.2 ± 194.9	96.3 ± 213.6	—	0.868
ALT, (U/L)	60.2 ± 97.4	60.6 ± 94.3	—	0.944
De Ritis ratio (2nd)	1.7 ± 1.7	1.8 ± 4.3	—	0.660
AST, (U/L)	66.7 ± 203.5	66.3 ± 139.4	—	0.969
ALT, (U/L)	78.6 ± 396.5	56.3 ± 208.3	—	0.178
ΔDDR	−0.03 ± 1.74	0.10 ± 4.32	—	0.539
GCS, median (IQR)	14 (8–15)	12 (7–15)	—	0.001
3–8	119 (27.2)	333 (36.6)	0.65 (0.51–0.83)	0.001
9–12	70 (16.0)	152 (16.7)	0.95 (0.70–1.30)	0.751
13–15	248 (56.8)	425 (46.7)	1.50 (1.19–1.88)	0.001
ISS, median (IQR)	20 (16–25)	22 (16–27)	—	0.004
1–15	50 (11.4)	66 (7.3)	1.65 (1.12–2.43)	0.010
16–24	226 (51.7)	440 (48.4)	1.14 (0.91–1.44)	0.248
≥25	161 (36.8)	404 (44.4)	0.73 (0.58–0.92)	0.009
Mortality, n (%)	46 (10.5)	88 (9.7)	1.10 (0.75–1.60)	0.623

ALT, alanine aminotransferase; AST, aspartate aminotransferase; CI, confidence interval; CVA, cerebral vascular accident; ΔDDR, delta De Ritis ratio (i.e., 2nd De Ritis ratio minus 1st De Ritis ratio); GCS, Glasgow Coma Scale; IQR, interquartile range; ISS, injury severity score; OR, odds ratio.

**Table 3:** Univariate and multivariate analysis of the risk factors for mortality of the patients.

	Univariate Analysis			Multivariate Analysis		
	OR	CI	*p*	OR	CI	*p*
Age, years	1.02	(1.01–1.03)	<0.001	1.03	(1.02–1.04)	<0.001
CVA, yes	2.07	(1.08–3.97)	0.029	2.20	(1.05–4.62)	0.037
HTN, yes	1.50	(1.04–2.15)	0.029	1.08	(0.70–1.66)	0.742
CAD, yes	2.17	(1.24–3.80)	0.007	1.45	(0.75–2.79)	0.272
ESRD, yes	6.04	(3.03–12.04)	<0.001	5.51	(2.48–12.25)	<0.001
GCS	0.87	(0.83–0.90)	<0.001	0.87	(0.83–0.91)	<0.001
ISS	1.07	(1.05–1.09)	<0.001	1.07	(1.04–1.09)	<0.001
ΔDDR, U/L	1.08	(1.01–1.15)	0.020	1.04	(1.01–1.08)	0.021

CAD, coronary artery disease; CI, confidence interval; CVA, cerebral vascular accident; ΔDDR, delta De Ritis ratio; ESRD, end-stage renal disease; GCS, Glasgow Coma Scale; ISS, injury severity score; HTN, hypertension; OR, odds ratio.

**Table 4:** Comparison of the injury, characteristics and outcomes of patients with a ΔDDR ≥0.7 vs. those with a ΔDDR < 0.7.

Variables	ΔDDR		OR (95% CI)	*p*
	≥0.7 *n* = 153	<0.7 *n* = 1194		
Female, n (%)	47 (30.7)	390 (32.7)	0.91 (0.64–1.32)	0.629
Age, years	61.9 ± 19.3	56.0 ± 19.2	—	<0.001
Diagnosis				
SAH, n (%)	64 (41.8)	534 (44.7)	0.89 (0.63–1.25)	0.498
SDH, n (%)	108 (70.6)	714 (59.8)	1.61 (1.12–2.33)	0.010
EDH, n (%)	22 (14.4)	187 (15.7)	0.90 (0.56–1.46)	0.680
ICH, n (%)	51 (33.3)	346 (29.0)	1.23 (0.86–1.75)	0.266
De Ritis ratio (1st)	1.7 ± 0.6	1.7 ± 0.7	—	0.961
AST, (U/L)	59.6 ± 69.2	100.2 ± 218.7	—	0.023
ALT, (U/L)	39.2 ± 45.6	63.2 ± 99.6	—	0.003
De Ritis ratio (2nd)	5.2 ± 10.2	1.3 ± 0.6	—	<0.001
AST, (U/L)	130.9 ± 290.3	58.2 ± 136.4	—	<0.001
ALT, (U/L)	93.5 ± 561.1	59.7 ± 224.4	—	0.165
Comorbidities				
CVA, n (%)	9 (5.9)	58 (4.9)	1.22 (0.59–2.52)	0.583
HTN, n (%)	77 (50.3)	391 (32.7)	2.08 (1.48–2.92)	<0.001
CAD, n (%)	15 (9.8)	78 (6.5)	1.56 (0.87–2.78)	0.133
CHF, n (%)	3 (2.0)	6 (0.5)	3.96 (0.98–16.00)	0.037
DM, n (%)	35 (22.9)	213 (17.8)	1.37 (0.91–2.05)	0.130
ESRD, n (%)	15 (9.8)	22 (1.8)	5.79 (2.94–11.43)	<0.001
GCS, median (IQR)	11 (6–15)	13 (7–15)	—	0.031
≤8	66 (43.1)	386 (32.3)	1.59 (1.13–2.24)	0.008
9–12	16 (10.5)	206 (17.3)	0.56 (0.33–0.96)	0.033
13–15	71 (46.4)	602 (50.4)	0.85 (0.61–1.19)	0.350
ISS, median (IQR)	25 (16–29)	20 (16–25)	—	0.001
1–15, n (%)	4 (2.6)	112 (9.4)	0.26 (0.09–0.71)	0.005
16–24, n (%)	58 (37.9)	608 (50.9)	0.59 (0.42–0.83)	0.002
≥25, n (%)	91 (59.5)	474 (39.7)	2.23 (1.58–3.14)	<0.001
Mortality, n (%)	48 (31.4)	86 (7.2)	5.89 (3.93–8.84)	<0.001
AOR of mortality	—	—	4.21 (2.68–6.63)	<0.001

ALT, alanine aminotransferase; AST, aspartate aminotransferase; CAD, coronary artery disease; CHF, congestive heart failure; CI, confidence interval; CVA, cerebral vascular accident; ΔDDR, delta De Ritis ratio (i.e., 2nd De Ritis ratio minus 1st De Ritis ratio); DM, diabetes mellitus; EDH, epidural hemorrhage; ESRD, end-stage renal disease; GCS, Glasgow Coma Scale; HTN, hypertension; ICH, intracerebral hemorrhage; IQR, interquartile range; ISS, injury severity score; OR, odds ratio; SAH, subarachnoid hemorrhage; SDH, subdural hemorrhage.

## Data Availability

No applicable.
